# Periodontitis and Metabolic Dysfunction-Associated Steatotic Liver Disease: emphasizing the clinical interplay between hepatologists and dentists

**DOI:** 10.1007/s10266-025-01184-4

**Published:** 2025-09-15

**Authors:** Nasser Mousa, Alaa Elmetwalli, Ahmed Abdel-Razik, Eman Mousa, Mostafa Abdelsalam, Sherif Elbaz, Niveen El-wakeel, Waleed Eldars, Elsayed Gad, Mona Arafa, Mohamed Abdelmaksoud, Mahmoud El-Bendary, Omar Marei, Ali El-Assmy, Aya Mousa, Adel El-Assmy, Gamal Shiha

**Affiliations:** 1 Tropical Medicine Department, Mansoura University, Mansoura, Egypt; 2 Egyptian Liver Research Institute and Hospital (ELRIAH), Mansoura, Egypt; 3 Prince Fahad bin Sultan Research Chair for Biomedical Research, University of Tabuk, Tabuk, Saudi Arabia; 4 Faculty of Dentistry, Mansoura University, Mansoura, Egypt; 5 Internal Medicine, Nephrology and Dialysis Unit, Mansoura University, Mansoura, Egypt; 6 Alameen General Hospital, Taif, Kingdom of Saudi Arabia; 7 Nephrology Fellow, HCA Houston Healthcare Kingwood/ Houston University, Texas, USA; 8 Medical Microbiology and Immunology Department, Mansoura National University, Gamasa City, Egypt; 9 Medical Microbiology and Immunology Department, Mansoura University, Mansoura, Egypt; 10 Medical Microbiology and Immunology Department, Faculty of Medicine, Delta University for Science and Technology, Mansoura, Egypt; 11 Faculty of Medicine, Mansoura University, Mansoura, Egypt; 12 Faculty of Physical Therapy, Galala University, Galala, Egypt; 13 Hepatology and Gastroenterology Unit, Internal Medicine Department, Mansoura University, Mansoura, Egypt

**Keywords:** MASLD, Metabolic-Associated Fatty Liver Disease, Periodontal disease, *Porphyromonas gingivalis*, Non-Alcoholic Fatty Liver Disease, Oral microbiota, Gut microbiota, Microbial dysbiosis

## Abstract

This narrative review aims to elucidate the connection between periodontitis and Metabolic Dysfunction-Associated Steatotic Liver Disease (MASLD), which is currently the most prevalent liver disease. By raising awareness of this link, healthcare providers can enhance their knowledge of the relationship between oral health and liver disease, offering valuable guidance to patients seeking advice from medical and dental professionals. A comprehensive search strategy was implemented to gather relevant literature from various databases, including PubMed, Scopus, and Web of Science. Keywords and controlled vocabulary terms related to MASLD, periodontitis, and their potential connections were used. Emphasis was placed on recent publications to incorporate up-to-date research findings. Various types of studies, such as primary research, systematic reviews, meta-analyses, guidelines, consensus statements, and expert opinions, were considered. The selection of studies focused on understanding the association between MASLD and periodontitis, as well as the underlying mechanisms. Special attention was given to studies exploring the oral–gut–liver axis and the pathogenic links between MASLD and periodontitis. Recent research suggests a possible connection between MASLD and periodontitis, as both conditions share common risk factors such as obesity, insulin resistance, and inflammation. Chronic inflammation associated with periodontitis may contribute to the progression of MASLD by promoting insulin resistance and systemic inflammation. The oral microbiome, which plays a crucial role in periodontitis development, may also influence MASLD pathogenesis. Dysbiosis in the oral microbiome can lead to the translocation of oral bacteria into the bloodstream, triggering systemic inflammation and potentially worsening liver damage in individuals with MASLD. Improving periodontal health can impact the progression of MASLD/MAFLD and enhance patient outcomes. Collaborative care models that integrate dental and medical expertise are crucial for optimal health outcomes in individuals with both conditions.

## Introduction

Around 25% of the global adult population is afflicted by Metabolic Associated Fatty Liver Disease (MAFLD), which was formerly known as Non-Alcoholic Fatty Liver Disease (NAFLD). This condition significantly burdens the health and financial systems of all societies [1, 2]. The classification of NAFLD has been a topic of debate in the medical community. Originally known as NAFLD, it was later renamed MAFLD and most recently Metabolic Dysfunction-Associated Steatotic Liver Disease (MASLD) to highlight the metabolic aspect of the condition. These terms are defined by diagnostic criteria related to metabolic risk factors. However, the differences in characteristics and mortality rates between NAFLD, MAFLD, and MASLD are still unclear. MASLD is the new term for this condition based on a recent multi-society Delphi meeting. With significant lifestyle changes over the past 2 decades, MASLD has become the most common liver disorder [3–7].

**Fig. 1:**
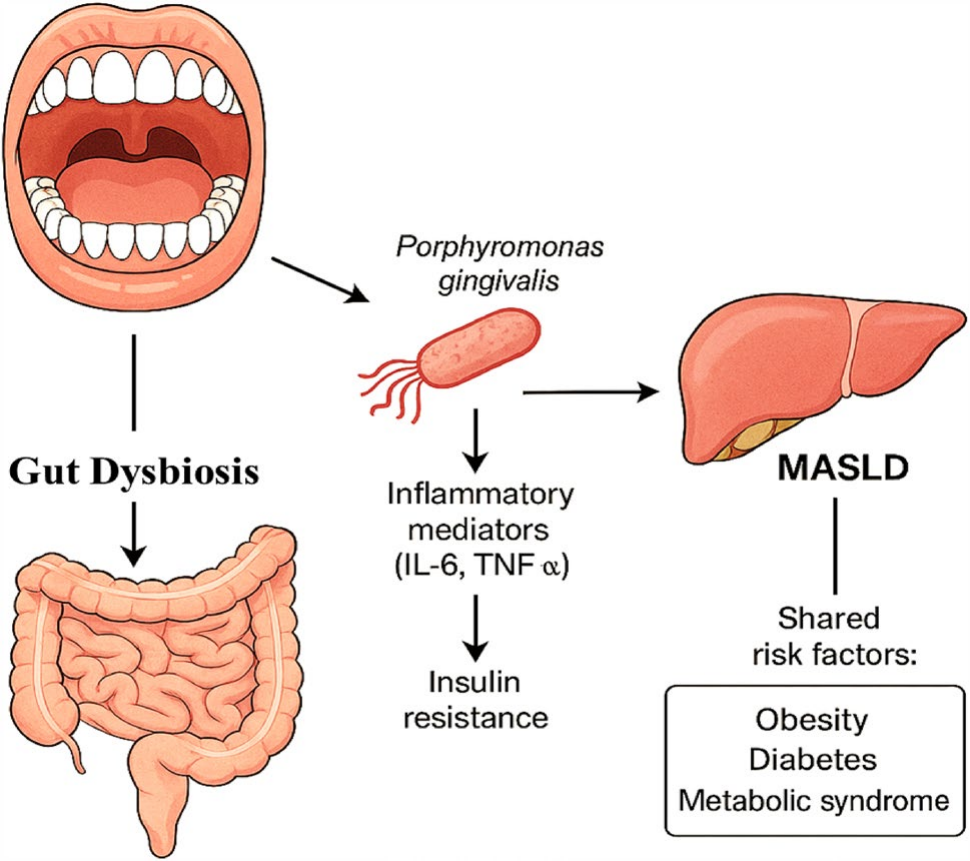
Interplay between periodontitis and MASLD through shared inflammatory and microbial pathways. The diagram illustrates how *Porphyromonas gingivalis* and other oral pathogens may contribute to gut dysbiosis, systemic inflammation, and insulin resistance—ultimately influencing MASLD development. Shared risk factors such as obesity, diabetes, and metabolic syndrome reinforce this bidirectional relationship

Periodontitis is a chronic, slow-progressing infectious condition of the tooth-supporting tissues that affects the underlying supporting tissues surrounding the teeth. Patients may develop gingivitis, periodontal attachment loss, alveolar bone resorption, and tooth loss. Periodontitis is a chronic, slow-progressing infectious condition that affects the supporting tissues surrounding the teeth. It is the sixth most common chronic disease worldwide, impacting nearly 750 million people. Beyond its oral manifestations, periodontitis has been increasingly recognized as a potential contributor to systemic diseases. Multiple studies have linked periodontal inflammation to diabetes, cardiovascular disease, chronic kidney disease, and liver conditions. Emerging evidence suggests a compelling interplay between periodontitis and MASLD, potentially mediated by shared risk factors such as obesity, insulin resistance, and chronic systemic inflammation. In addition, the oral–gut–liver axis—through microbial translocation and immune modulation—may serve as a key mechanistic pathway bridging these conditions. This complex network is illustrated in Fig. 1, highlighting the microbial and inflammatory pathways linking oral and hepatic health. Recognizing this bidirectional relationship underscores the need for a multidisciplinary approach, encouraging collaborative care between hepatologists and dental professionals to optimize prevention and management strategies [8, 9].

Considerable advancements have been made in periodontal medicine in recent years, and presently, over 50 systemic illnesses are under scrutiny for their potential link to periodontal disorders. Numerous research findings have indicated that periodontal ailments are correlated with heightened susceptibility to several systemic infections, including diabetes mellitus, cardiovascular disease, liver diseases, chronic obstructive pulmonary disease, chronic kidney disease, and cancer [10–12]. A 2012 workshop by the European Federation of Periodontology and the American Academy of Periodontology reviewed links between periodontitis and systemic diseases, including cardiovascular issues. An updated consensus in 2019 added more studies [13].

Several theories indicate a connection between fatty liver disease (FLD) and periodontitis via the presence of periodontal pathogens, inflammatory cytokines, and oxidative stress [14]. There is compelling evidence to suggest that *Porphyromonas gingivalis* (PG), a leading factor in causing periodontitis, alters the makeup of gut microflora. This modification has a significant impact on the pathogenesis of FLD. Compared to non-FLD controls, the prevalence of *P. gingivalis* was higher in individuals with FLD [15]. Moreover, individuals who had experienced periodontal clinical attachment loss exhibited a higher incidence of FLD [16]. Researchers have discovered a notable correlation between periodontitis and liver steatosis, especially in individuals who have progressed to liver disorders [17].

Furthermore, PG was observed in patients with FLD who also had lower serum albumin levels, although this association may be influenced by nutritional status or other confounding factors. Moreover, infection could be an independent predictor of FLD development, and persistent PG infection can speed up liver tissue fibrosis and lower liver function [18, 19]. Therefore, this article presents a narrative review of the current literature exploring the biological and clinical connections between periodontitis and MASLD, with particular attention to shared mechanisms and implications for interdisciplinary care.

### Literature selection approach

This review is based on a comprehensive search of the literature conducted using PubMed, Scopus, and Web of Science up to June 2024. Keywords and MeSH terms included “periodontitis,” “MASLD,” “MAFLD,” “NAFLD,” “oral microbiome,” “gut-liver axis,” and “systemic inflammation.” Articles were selected based on relevance, recency, and scientific quality. Inclusion criteria focused on peer-reviewed English-language articles addressing the relationship between periodontitis and MASLD/MAFLD. Editorials, case reports, conference abstracts, and non-human studies not directly related to human pathophysiology were excluded. Although most studies indicated a positive association, a few reported no significant relationship; these perspectives were reviewed for balance.

## From the NAFLD–MAFLD era to the MASLD era

**Fig. 2:**
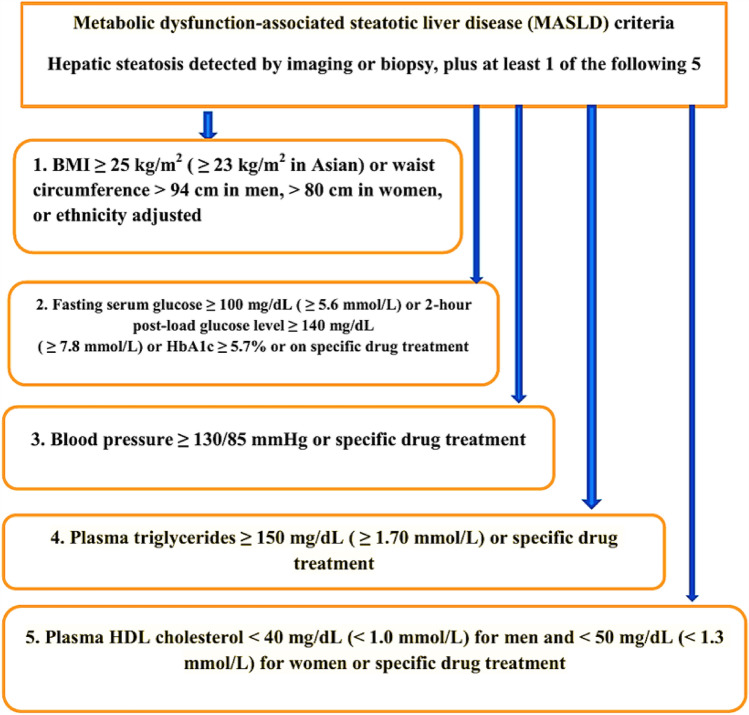
Updated diagnostic criteria for MASLD, reflecting the recent nomenclature change from NAFLD/MAFLD. Diagnosis requires evidence of hepatic steatosis by imaging or biopsy, plus at least one of five metabolic risk factors: obesity (or increased waist circumference), impaired glucose metabolism, elevated blood pressure, hypertriglyceridemia, or low HDL cholesterol. This framework highlights the central role of metabolic dysfunction in MASLD pathogenesis

FLD poses a significant challenge to public health on a global scale. The categorization of FLD is dependent on the individual’s alcohol consumption history, distinguishing it into alcoholic fatty liver disease (ALD) and NAFLD [20]. This categorization is imperfect because people with NAFLD tend to consume moderate amounts of alcohol, and those categorized as ALD and consuming dangerous quantities of alcohol have a rising incidence of metabolic dysfunction. In addition, to define NAFLD accurately, other potential factors leading to liver disease must be eliminated from consideration. As a result, the collective impact of several factors on the advancement of illnesses often goes unnoticed, leading to the exclusion of a significant number of patients from clinical trials within this domain [21, 22]. In 2020, international experts recommended renaming NAFLD in adults to MAFLD [23]. In 2023, three major liver associations suggested replacing NAFLD with MASLD **(**Fig. 2**)** and non-alcoholic steatohepatitis with metabolic dysfunction-associated steatohepatitis (MASH) [4]. Studies show a high agreement between NAFLD and MASLD definitions, with approximately 99% overlap [24]. This updated terminology better reflects the underlying mechanisms for this prevalent liver condition [4]. However, the terms MAFLD and MASLD have more in common than not, and are more appropriate for the condition [25]. The Delphi consensus recommended renaming NAFLD or MAFLD to MASLD. This change simplifies diagnosis by removing the need for high-sensitivity C-reactive protein or HOMA-IR assessments, which are often not available in routine clinical practice. This is especially beneficial for lean patients [26].

## Association of NAFLD/MASLD and periodontitis

Since 2010, studies on the link between NAFLD and periodontal disease have drawn global attention. Experts now recommend the term MAFLD instead of NAFLD. Implementing MALD/MASLD terminology and definition will increase the diagnosis of chronic liver disease, thus emphasizing the importance of periodontitis in chronic liver disease. NAFLD was observed in 2,623 participants with periodontal disease. After 7.7 years, adjusting for risk factors, NAFLD incidence was moderately higher (ratio of 1.60) in those with ≥ 30% of periodontal sites with clinical attachment level ≥ 3mm, compared to healthy periodontium participants [27]. Kuroe et al. investigated how periodontitis affects the advancement of hepatic fibrosis in 341 Japanese patients with NAFLD who did not have hepatic fibrosis when their observation period began. Upon a 5-year observation, the authors discovered that patients afflicted with moderate to severe periodontitis are more likely to develop hepatic fibrosis, with an odds ratio of 2.06 (0.89–4.76) [28]. During a 12-year investigation involving 6,165 individuals, Helenius-Hietala and her team determined that the presence of advanced periodontitis and an NAFLD diagnosis at the beginning of the study resulted in an increased probability of severe liver complications, including liver-related hospitalization and hepatocellular carcinoma [29]. A correlation was discovered between periodontal pockets and both hepatic inflammation and symptoms of metabolic syndrome, indicating a positive relationship [30]. Two Korean studies found a connection between fatty liver index and periodontal disease in 4000 people [31, 32]. Alazawi et al. showed increased periodontitis prevalence in NASH/fibrosis patients based on liver biopsy [33]. Akinkugbe et al. found a link between periodontitis, NAFLD prevalence, and high CRP levels [16]. Two reviews and meta-analyses found 12 studies indicating a link between periodontal disease and NAFLD, with periodontitis linked to NAFLD and cirrhosis. Patients with periodontal disease showed greater odds ratios for NAFLD and cirrhosis. Furthermore, tooth loss is related to NAFLD [34, 35]. This shows an association between MAFLD and periodontitis.

## Oral–gut–liver axis

The mouth and gut, including the liver, are linked by the gastrointestinal tract, with saliva and digested food acting as a chemical connection. The oral mucosa protects against microbes and toxins from the environment. Microorganisms in the mouth can be beneficial or harmful and are regulated by protective mechanisms [36]. The organisms residing in the mouth are crucial in maintaining homeostasis and preventing disease. Various factors may disrupt the balance of microorganisms in the mouth, leading to oral dysbiosis. These factors may lead to a decline in the functioning of salivary glands, a decrease in the ability to clear oral debris, a shift towards a more acidic oral environment, Gingival inflammation, and alterations in the levels of immunoglobulin A present in saliva [37]. The red complex bacteria (specifically *Porphyromonas gingivalis*, *Treponema denticola*, and *Tannerella forsythia*) play a crucial role in the development of periodontitis, with considerable quantities of these bacteria found in both active periodontal lesions and periodontal pockets [38]. It is effortless for these particular periodontal microorganisms to travel from the mouth into the gut through swallowing, potentially disrupting the balance of the gut microbiome [39]. There is a growing body of evidence suggesting that an imbalance in the microbiota of the intestines caused by periodontal bacteria obtained through oral means may serve as a connecting factor linking periodontitis and systemic illnesses. Recent research indicates liver disease is strongly associated with gut microbiota and oral microbiota imbalance. Furthermore, several studies have proven that individuals suffering from chronic liver disease exhibit imbalanced oral microbial dysbiosis [40, 41].

## Pathogenic link between MASLD/ MAFLD and periodontitis

The influence of periodontal inflammation has been documented in many systemic diseases. The change in terminology from NAFLD to MASLD/ MAFLD increases the spectrum of diseases under the umbrella of FLD compared to NAFLD and consequently increases the link between periodontitis, liver diseases, and metabolic disturbance. However, the precise connection between MASLD/MAFLD and periodontitis remains uncertain. One probable explanation is the worsening of insulin resistance (a key event in MASLD/MAFLD). Periodontitis-induced systemic inflammation could potentially result in insulin resistance due to elevated levels of adipocytokines in the bloodstream, such as cytokines like IL-6, leptin, and tumor necrosis factor alpha, which possess the ability to inhibit the insulin receptor and its subsequent signaling activities [42, 43]. In addition, periodontal bacteria, especially *P. gingivalis*, existing in dental plaque, have numerous virulence factors, such as lipopolysaccharide, fimbriae, and enzymes, which can produce inflammation in periodontal tissues. The raised lipopolysaccharides and proinflammatory cytokines initiated by these oral bacteria have been reported to induce and worsen insulin resistance. Moreover, in vivo studies declared that *P. gingivalis* can encourage the build-up of fat, raise the immune reaction, and trigger insulin resistance, ultimately leading to the progression of FLD [44].

Another possible mechanism that links periodontitis and MASLD/MAFLD is induced gut dysbiosis by pathogenic oral microbiota. Periodontitis is characterized by salivary oral dysbiosis that is involuntarily swallowed with saliva. This swallowed pathogenic oral microbiota induces gut dysbiosis [45]. Also, in the case of oral dysbiosis, the hematogenous systemic spread of oral bacteria and their toxins from local oral lesions is related to this scenario [46, 47]. Evidence indicates intestinal dysbiosis prompted by bacteria that cause periodontal disease is strictly linked to decreased expression of tight junction proteins, increased expression of genes related to inflammatory cytokines, and a subsequent inflammatory response in the liver tissue. This implies that alterations in the gut microbiota triggered by oral bacteria play a role in the development and advancement of FLD, as these inflammatory modifications are linked to the condition [48].

An additional mechanism is increasing evidence that oral dysbiosis in periodontal disease induces a systemic inflammatory milieu that contributes to liver diseases, including MASLD/MAFLD [39–41]. Furusho et al. discovered that intriguing PG infection led to faster liver inflammation and fibrosis in mice with fatty liver [49]. *Porphyromonas gingivalis* and bacterial agents like LPS and cytokines can spread to the liver by hematogenous dissemination of periodontal bacteria via the micro-circulation in the periodontal pocket during regular daily events, such as brushing and entering the hepatic circulation through the hepatic artery or direct swallowing of periodontal bacteria via the oro-digestive route and its colonic colonization inducing intestinal dysbiosis [45, 50].

## Systemic and immune factors correlating MASLD/MAFLD and periodontitis

Inflammation in one organ can affect neighboring and distant tissues through circulating inflammatory mediators. The reclassification of NAFLD to MASLD/MAFLD potentially strengthens the connection between periodontitis and liver diseases. However, the exact relationship between FLD and periodontitis remains unclear. Despite appearing as a localized infection, periodontal pathogens and their by-products can enter the bloodstream, contributing to systemic complications [51]. In periodontitis, pathogen-associated molecular patterns are released during infection, triggering an inflammatory response by binding to host cell receptors. This inflammatory response can lead to increased expression of inflammatory mediators, such as cytokines and chemokines, which can exacerbate hepatic inflammation in FLD. Adhesion molecules also play a role in recruiting immune cells to the infected area and linking periodontitis to systemic complications, including the MASLD/ MAFLD [52].

**Fig. 3:**
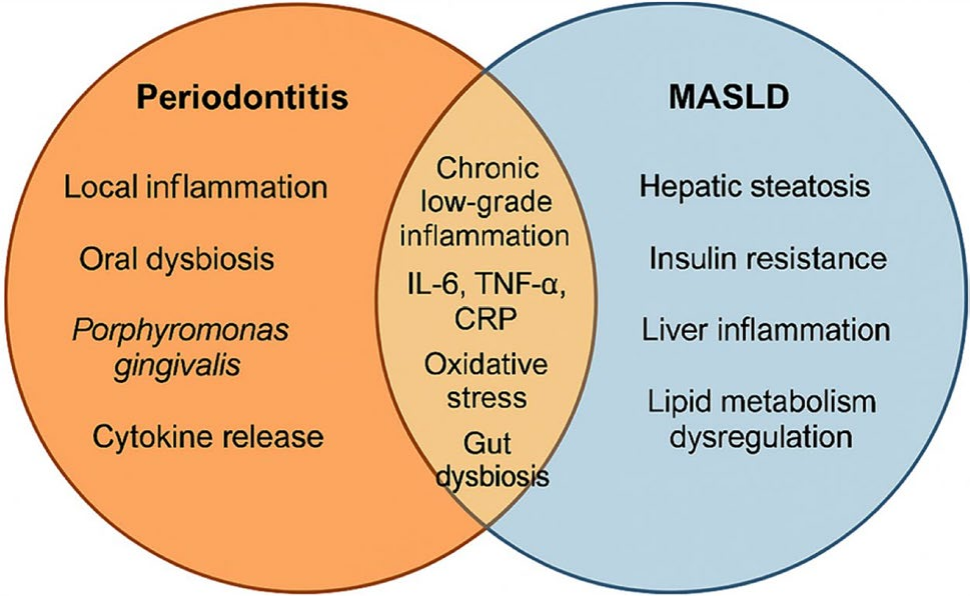
Shared metabolic pathways linking periodontitis and MASLD. Both diseases are influenced by overlapping metabolic conditions—obesity, insulin resistance, diabetes, hypertension, and dyslipidemia—which contribute to systemic inflammation, immune dysregulation, and gut–liver axis disruption

Furthermore, periodontitis and liver disease can trigger inflammatory responses and produce inflammatory mediators that can affect each other. Cirrhotic patients have higher levels of cytokines such as IL-1α, IL-1β, TNF-α, and IL-6, which drive inflammation, steatosis, fibrosis, and cancer development. Elevated levels of these cytokines can contribute to periodontal tissue destruction by increasing collagenase and MMPs activity. Cirrhosis also impairs immune responses, leading to immune dysfunction or immune paralysis, potentially increasing the risk of periodontitis in cirrhotic patients [53]. Conversely, advanced periodontitis can worsen liver health by causing systemic inflammation, which can impact the prognosis of liver cirrhosis (Fig. 3). This indicates a two-way negative connection between periodontal and liver disease due to their inflammatory nature [54]. Th17 may be a key molecule for explaining the relationship between periodontal disease and FLD. Infection with *P. gingivalis* can activate the Th17 axis, which worsens with liver steatosis progression, potentially impacting hepatitis and fibrosis. Also, Th17 cells from periodontitis can migrate to the gut and liver, worsening FLD. In addition, IL-17 contributes to intestinal barrier dysfunction.

Severe periodontitis affects both cytotoxic and helper T cells, with an increase in central memory T cells. While the total number of CD4 + T cells is higher in severe periodontitis, their ability to produce IFN-γ is reduced, and the number of CD4 + IFN-γ + cells in circulation is decreased in these patients [55]. In addition, periodontal disease is characterized by an inflammatory state, with increased levels of proinflammatory cytokines like tumor necrosis factor alpha and IL-6. This can lead to a higher risk of MASLD/MAFLD. Tumor necrosis factor alpha can cause hepatic insulin resistance by blocking insulin receptor signaling. IL-6, activated by tumor necrosis factor alpha, also affects insulin signaling and promotes fatty acid oxidation and C-reactive protein secretion by the liver [56]. Moreover, cytokines and chemokines released during periodontal inflammation can enter systemic circulation, potentially exacerbating hepatic inflammation in MAFLD [42, 43].

## Shared metabolic pathways in periodontitis and MASLD

**Fig. 4:**
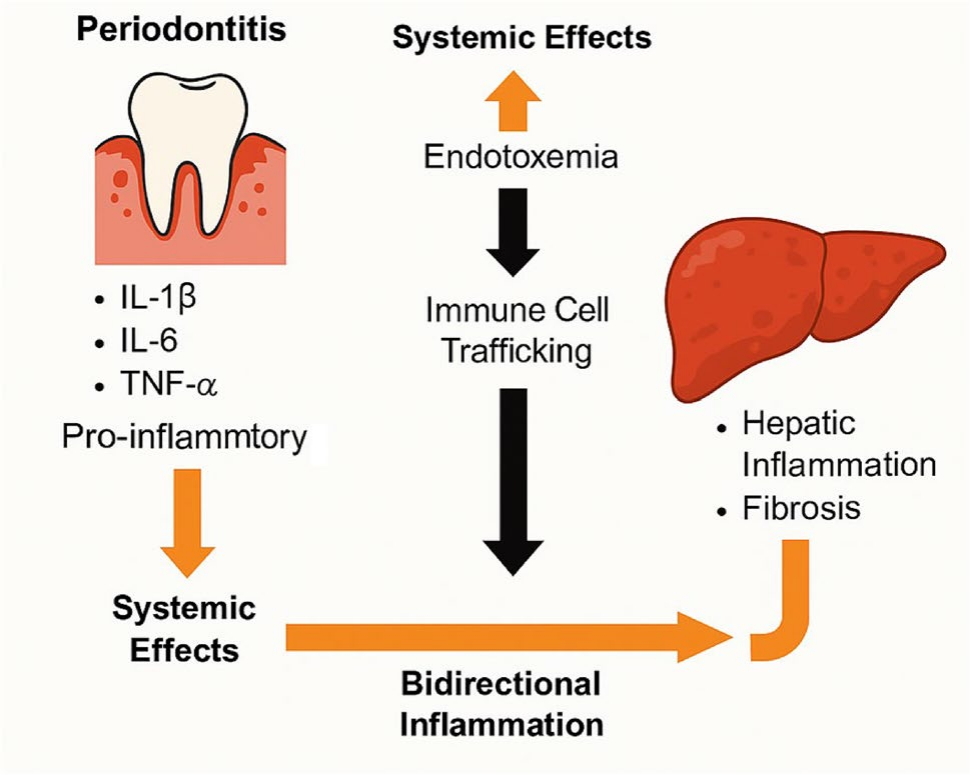
Systemic immune and inflammatory crosstalk between periodontitis and MASLD. Periodontitis leads to the release of proinflammatory cytokines (IL-1β, IL-6, TNF-α), contributing to systemic effects such as endotoxemia and immune cell trafficking. These systemic responses can exacerbate hepatic inflammation and fibrosis. In turn, MASLD-associated inflammation may contribute to systemic immune dysregulation, highlighting a bidirectional relationship driven by chronic inflammation

MASLD is fundamentally defined by the coexistence of hepatic steatosis and one or more metabolic dysfunctions such as obesity, type 2 diabetes mellitus, arterial hypertension, or dyslipidemia. Interestingly, these same metabolic abnormalities are also closely linked to the pathogenesis and severity of periodontitis. This overlap suggests that both diseases may be interconnected through shared systemic pathways involving chronic inflammation, immune dysregulation, oxidative stress, and gut microbial imbalance. In the following subsections, we explore how these individual metabolic disorders serve as common risk factors and mechanistic bridges between MASLD and periodontitis. Figure 4 provides a visual summary of these shared pathophysiological mechanisms. To better understand this interconnected framework, each of the following subsections illustrates how individual metabolic components central to MASLD also contribute mechanistically to periodontal disease, reinforcing the systemic interplay between oral and hepatic inflammation.

### Obesity as a common driver of both periodontitis and MASLD

Obesity contributes to both periodontitis and MASLD through chronic systemic inflammation, adipokine imbalance, and immune dysregulation. It is a major diagnostic component of MASLD and an established risk factor for periodontal tissue destruction. Overweight/obesity is a major component in MASLD/ MAFLD diagnosis [4, 21, 22]. The 2017 periodontal disease classification system indicates that obesity is a significant metabolic dysfunction that substantially impacts the state of periodontal health, predominantly contributing to the progression and exacerbation of periodontal inflammation [56]. In 1998, Saito et al. conducted a cross-sectional study with 241 Japanese people, which marked the first instance of a connection being established between obesity and periodontal disease in humans. After that, various epidemiological studies have confirmed the speculation that obesity constitutes a hazard for periodontitis [57].

Furthermore, Wood and colleagues established a connection between periodontal disease and body composition by analyzing statistics from the Third National Health and Nutrition Examination Survey. Over the past 10 years, there has been a substantial increase in the literature concerning the correlation between obesity and periodontal disease [58]. The higher possibility of periodontitis in obesity suggests that there is a correlation between obesity and periodontitis that leads to the presence of comorbidities. It was found that obesity results in an inflammatory setting and disrupts bone homeostasis as the primary consequence [59]. Studies have consistently demonstrated that obesity may worsen alveolar bone loss in periodontitis and decrease the height of the alveolar bone crest. This suggests that obesity could be considered a potential risk factor, even in cases where the periodontium is clinically healthy [60, 61]. The fundamental processes responsible for the association between obesity and periodontitis are yet to be understood. Various factors could explain the connection between these two conditions, such as inflammation, bone activity changes, genetics, microbial population imbalances, high metabolism, and alterations in the bone marrow environment [62]. Also, obesity may be linked to periodontitis via immune system impairment and increased susceptibility to infectious diseases due to a hyper-inflammatory state. Adipose tissue can release cytokines, adipokines, and C-reactive proteins that contribute to chronic inflammation and increased tissue breakdown in obesity. On the other hand, in periodontal disease, cytokines can increase adipose inflammation, leading to higher levels of advanced glycation end products and reactive oxygen species that may harm periodontal tissues [63, 64].

### The bidirectional relationship between diabetes, periodontitis, and MASLD

Type 2 diabetes is a core criterion for MASLD diagnosis and is also tightly linked to periodontal disease via insulin resistance and proinflammatory cytokines. This shared inflammatory profile helps explain the interplay between liver and periodontal health. Diabetes mellitus is a key component in MASLD/ MAFLD diagnosis [4, 21, 22]. Diabetes and periodontitis have a bidirectional relationship [65]. Essentially, severe periodontitis significantly heightens the chances of developing diabetes, while diabetes is a major contributing factor in the occurrence of severe periodontitis [65, 66]. People with diabetes have a 2–3 times higher risk of periodontitis than non-diabetics, especially with poor glycemic control. Periodontitis is linked to higher HbA1c and glucose levels and a greater risk of diabetes in non-diabetic people.

Furthermore, diabetic patients with periodontitis are at higher risk for complications [67, 68]. Various theories exist regarding the association between diabetes and periodontitis, yet they are still debated. Periodontal infection by *P. gingivalis* leads to elevated levels of lipopolysaccharide, TNF-α, and IL-6, inducing acute phase proteins like CRP. These cytokines, produced locally in inflamed periodontal tissues, enter the circulation and increase the risk of βeta cell dysfunction, insulin resistance, impaired glucose uptake, higher HbA1c, and worsening glycemic control in normoglycemic and hyperglycemic individuals. Also, cytokines affect insulin and GLUT receptors, reduce insulin secretion, increase resistance, and cause hyperglycemia [69, 70]. Hyperactive neutrophils in periodontium produce reactive oxygen species, activating proinflammatory pathways and insulin resistance in patients with periodontitis and diabetes [71]. Increased oxidative stress induces lipid peroxidation, having a proinflammatory effect. A study found that diabetes patients had higher gingival crevicular fluid (GCF) markers of lipid peroxidation, correlating with periodontitis clinical parameters and inflammatory mediator levels [72].

On the other hand, diabetes raises the risk of periodontal disease due to inflammation in the periodontal tissues caused by chronic hyperglycemia. Chronic hyperglycemia has been found in studies to create advanced glycation end products (AGEs) that can attach to particular receptors (RAGE) on various cells such as macrophages, fibroblasts, and endothelial cells, consequently promoting heightened levels of inflammation, oxidative stress, localized tissue harm, and eventually bone deterioration [73]. As a result, macrophages become hyperactive, producing proinflammatory cytokines such as IL-1, IL-6, and TNF-α. AGEs can also cause endothelial cells to become more permeable and express more adhesion molecules, while fibroblasts produce less collagen and negatively impact bone metabolism, leading to weakened repair and formation [74, 75]. The theory that RAGE activation contributes to the etiology of periodontitis in diabetic individuals was supported by Lalla et al. In diabetic gingiva, AGE build-up is increased and interacts with RAGE to produce excessive amounts of proinflammatory cytokines, cause vascular dysfunction, and loss of effective tissue integrity and barrier function [76].

Furthermore, AGEs can accumulate on either mononuclear or polymorphonuclear cells, suppressing their chemotactic and phagocytic abilities. This ultimately facilitates the progression of Gram-negative anaerobic bacteria (the most prevalent bacterial flora associated with periodontitis), which may clarify the increased occurrence and seriousness of periodontal disease in individuals with diabetes [77].

### Hypertension and endothelial dysfunction in periodontitis and MASLD

Both periodontitis and MASLD are associated with endothelial dysfunction and increased vascular inflammation. Hypertension serves as both a complication and a shared consequence, reinforcing the systemic nature of the oral–liver link. Periodontitis has been suggested to be a modifiable, non-traditional risk factor that considerably heightens the likelihood of cardiovascular diseases [78, 79]. This presents a new perspective on the ailment, considering its possible impact on health beyond oral discomfort. In 2019, the World Heart Federation and the European Federation of Periodontology reached a consensus stating that treating periodontitis effectively can improve cardiovascular disease (CVD) progression. In addition, it has been suggested that high-risk patients and those with established CVD should actively manage traditional cardiovascular risk factors, such as hypertension, in the presence of periodontitis [80].

Both hypertension and periodontitis affect nearly one-third of adults internationally, particularly older age, men, smokers, overweight/obese persons, diabetics, and those of low socioeconomic status [81]. Generally, a person with periodontitis presents with increased blood pressure and has a nearly 30–70% chance of developing hypertension. A large meta-analysis of 40 studies found that periodontitis increases the risk of developing arterial hypertension, with observational studies showing a positive correlation between periodontitis and higher blood pressure [82]. Furthermore, periodontitis reduces the effectiveness of antihypertensive therapy, and the treatment of periodontitis lowers blood pressure in hypertensive patients [83]. Hypertension in periodontitis is linked to endothelial dysfunction and impaired vasodilatation. Periodontitis is known to reduce the effectiveness of vasodilatation, which is dependent on the endothelium. This is caused by the rise of inflammatory biomarkers (CRP and IL-6) in the body, which negatively affect the lipid profile, generate more superoxide radicals in the vascular system, and impede the expression of vascular nitric oxide synthase-3 (NOS-3) [84, 85]. Oral bacteria may cause periodontitis and elevated blood pressure by decreasing nitric oxide production, leading to vascular dysfunction [86]. Also, animal research indicates that the immune response to *P. gingivalis* triggers high blood pressure, inflamed blood vessels, and impaired vessel lining function [87]. The chemotactic activity of immune cells, such as lymphocytes and monocytes, may be heightened by periodontal inflammation. These cells are responsible for the production of cytokines (TNF-α, IL-6, and IL-17), which lead to vascular impairment, acceleration of atherosclerosis, and elevated blood pressure [88, 89].

### Dyslipidemia and lipid metabolism disturbances linking MASLD and periodontitis

Dyslipidemia promotes hepatic steatosis and also influences periodontal breakdown via oxidative stress and altered immune responses. These lipid-driven pathways represent another mechanism connecting MASLD and oral inflammation. Studies suggest a possible correlation between dyslipidemia and periodontitis [90, 91]. Periodontitis and hyperlipidemia are connected through common mechanisms like LPS responses, genetics, smoking, and stress [92]. Periodontitis causes proinflammatory cytokines, leading to systemic inflammation and a bidirectional relationship with dyslipidemia. Likewise, hyperlipidemia deregulates immune cells and wound healing, increasing susceptibility to periodontitis. Conversely, research indicates that periodontitis increases serum lipid levels via the systemic effects of proinflammatory cytokines (TNF-α/IL-1β). This exacerbates the deterioration of periodontal tissue reaction [93, 94] . According to Iacopino and Cutler, a rise in proinflammatory cytokine levels in response to chronic periodontitis elevates blood lipid levels. Infection with Gram-negative periodontal bacteria may result in systemic IL-1 and TNF-α production, leading to chronic hypertriglyceridaemia [95]. Periodontitis and accompanying oral dysbiosis can cause dyslipidemia by raising local and systemic inflammatory molecules and cytokines. These inflammatory reactions and infections are accompanied by increased triglyceride and LDL cholesterol production and hepatic HDL cholesterol breakdown [96, 97]. It also plays a role, as demonstrated by the correlation between periodontal status and plasma levels of anti-oxidized-LDL-C antibodies [98]. Good oral hygiene and regular tooth brushing can improve dyslipidemia, especially high-density lipoprotein cholesterol and triglyceride levels [99].

## Effect of recent therapies suggested to reverse MASLD/MAFLD

The recent FDA approval of resmetirom offers hope for treating non-cirrhotic MASLD with promising results in clinical trials. Resmetirom targets the liver and has shown effectiveness in reducing hepatic fat, improving liver health, and reducing biomarkers of liver damage without affecting weight or glucose metabolism [100]. Further, anti-diabetic medications such as SGLT2 inhibitors and GLP-1RAs are being studied for their potential to reverse liver steatosis and prevent severe fibrosis [101]. Treatment with GLP-1Ras not only improves body weight and glycemic control but also enhances cardiometabolic parameters, such as lowering blood pressure, improving lipid profile, and reducing proteinuria. These benefits make GLP-1Ras a desirable option for physicians, particularly in managing patients with MAFLD and diabetes [102, 103]. In contrast, SGLT2 inhibitors seem to be effective in reversing metabolic and hepatic abnormalities linked to MAFLD. However, till now, no study has focused on the impact of these new drugs on periodontitis.

## Clinical implications and interdisciplinary care

**Fig. 5:**
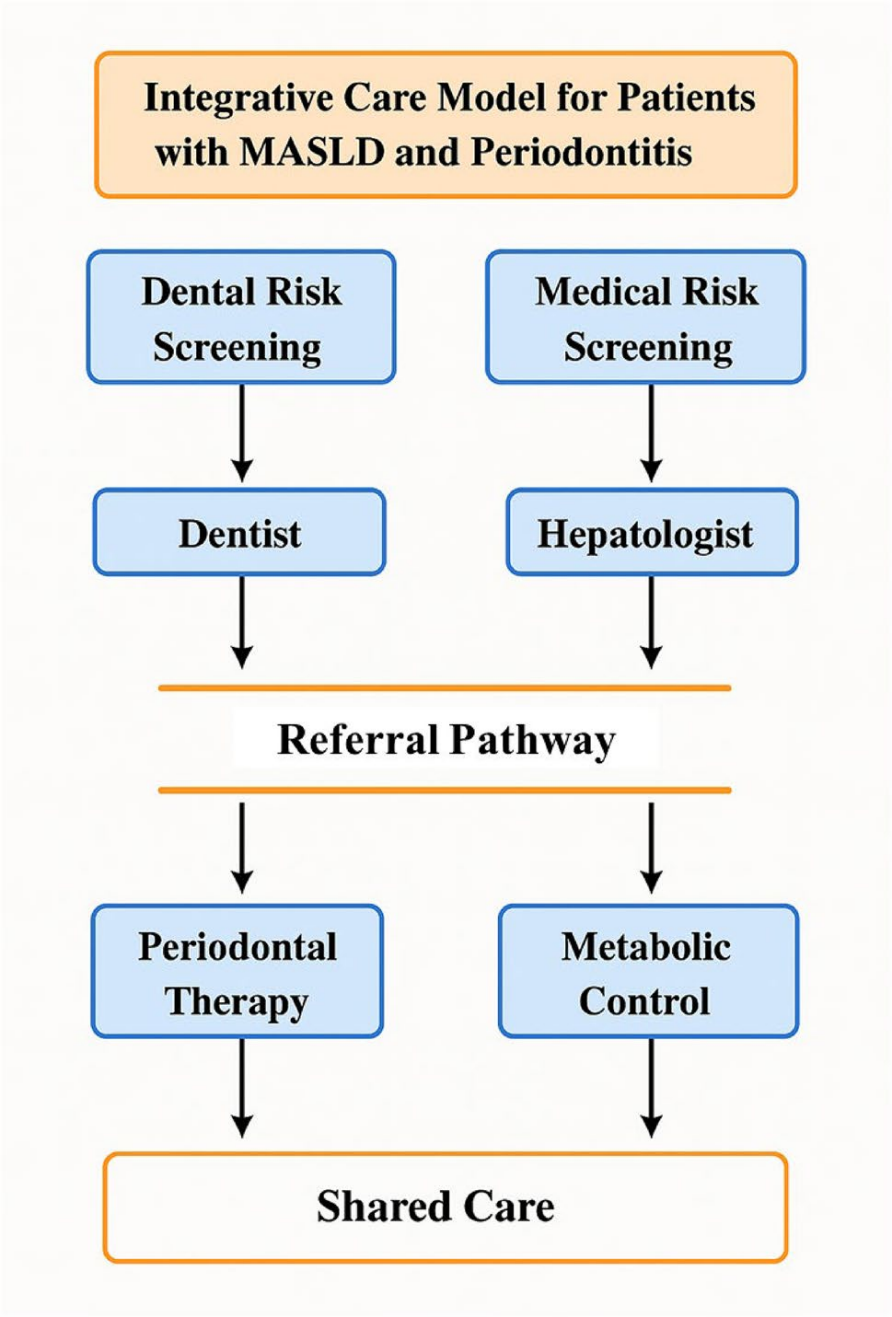
Integrative care model for patients with MASLD and periodontitis. The workflow emphasizes early risk screening in both medical and dental settings, bidirectional referral between hepatologists and dental professionals, and coordinated treatment that combines periodontal therapy with metabolic disease management. This interdisciplinary approach supports better outcomes through systemic inflammation control and shared care protocols

The growing body of evidence connecting periodontitis and MASLD highlights the urgent need for integrated care models that bridge dental and medical practice. Traditional healthcare silos often overlook the systemic effects of oral disease, especially in patients with metabolic dysfunction. As illustrated in Fig. 5, a collaborative approach should begin with mutual risk screening in both settings—dentists identifying patients with metabolic risk factors, and hepatologists recognizing oral health as a potential contributor to systemic inflammation. Bidirectional referral between dental professionals and hepatologists is essential to ensure early detection, comprehensive evaluation, and timely intervention. Shared care plans involving periodontal therapy, nutritional guidance, and metabolic disease management can reduce inflammatory burden, improve quality of life, and potentially mitigate progression of both diseases. Embedding oral health into chronic disease management may serve as a cost-effective and clinically impactful strategy in the era of MASLD.

## Conclusion

There is growing evidence that periodontitis may contribute to the development and progression of MASLD through systemic inflammation, immune modulation, and alterations in the oral–gut–liver axis. Addressing periodontal health could serve as a complementary strategy in managing MASLD by reducing systemic inflammatory burden and metabolic stress. These findings support the importance of integrated care models that foster collaboration between hepatologists and dental professionals. Further research is needed to explore the potential bidirectional nature of this relationship and to clarify whether MASLD also exerts a significant influence on periodontal health.

## Data Availability

The datasets used and/or analyzed during the current study are available from the corresponding author upon reasonable request.

## References

[1] Younossi Z, Anstee QM, Marietti M, Hardy T, Henry L, Eslam M, et al. Global burden of NAFLD and NASH: trends, predictions, risk factors and prevention. Nat Rev Gastroenterol Hepatol. 2018;15:11–20. 28930295 10.1038/nrgastro.2017.109

[2] Kaya E, Zedginidze A, Bechmann L, Canbay A. Metabolic dysfunction-associated fatty liver disease (MAFLD) and non-alcoholic fatty liver disease (NAFLD): distinct fatty liver entities with different clinical outcomes? Hepatobiliary Surg Nutr. 2022;11(2):299–301. 35464288 10.21037/hbsn-21-548 PMC9023843

[3] Ramírez-Mejía MM, Jiménez-Gutiérrez C, Eslam M, et al. Breaking new ground: MASLD vs. MAFLD—which holds the key for risk stratification? Hepatol Int. 2024;18:168–78. 38127259 10.1007/s12072-023-10620-y

[4] Rinella ME, Lazarus JV, Ratziu V, Francque SM, Sanyal AJ, Kanwal F, et al. A multi society Delphi consensus statement on new fatty liver disease nomenclature. J Hepatol. 2023;S0168–8278(23):00418. 10.1097/HEP.0000000000000696 37983810

[5] Wong VW, Wong GL, Woo J, Abrigo JM, Chan CK, Shu SS, et al. Impact of the new definition of metabolic associated fatty liver disease on the epidemiology of thedisease. Clin Gastroenterol Hepatol. 2021;19(10):2161-71 e5. 33137486 10.1016/j.cgh.2020.10.046

[6] Mousa N, EL-Eraky A, Arafa M, Elalfy H, Farag R, Abdel-Razik A, et al. Value of hepatic artery resistive index in evaluation of liver fibrosis related to non-alcoholic fatty liver diseases. Med J Viral Hepatitis. 2023;7(2):1–8.

[7] Ahmed Ezzat A, Elmoghazy M. Metabolic dysfunction-associated fatty liver disease from definition to complications. Med J Viral Viral Hepatitis. 2021;6(1):4–9.

[8] Hassan E, Abdel-Razik A, Eman M, et al. Periodontal disease as predictor of chronic liver diseases. Med J Viral Viral Hepat. 2020;4(2):57–66.

[9] Kinane DF, Stathopoulou PG, Papapanou PN. Periodontal diseases. Nat Rev Dis Primers. 2017;3:17038. 28805207 10.1038/nrdp.2017.38

[10] Fi C, Wo W. Periodontal disease and systemic diseases: an overview on recent progresses. J Biol Regul Homeost Agents. 2021;35:1–9. 33463138

[11] Abnet CC, Qiao YL, Mark SD, Dong ZW, Taylor PR, Dawsey SM. Prospective study of tooth loss and incident esophageal and gastric cancers in China. Cancer Causes Control. 2001;12:847–54. 11714113 10.1023/a:1012290009545

[12] Heikkila P, But A, Sorsa T, Haukka J. Periodontitis and cancer mortality: register-based cohort study of 68,273 adults in 10-year follow-up. Int J Cancer. 2018;142:2244–53. 29322513 10.1002/ijc.31254

[13] Sanz M, Marco Del Castillo A, Jepsen S, et al. Periodontitis and cardiovascular diseases: consensus report. J ClinPeriodontol. 2020;47(3):268–88. 10.1111/jcpe.13189 PMC7027895 32011025

[14] Han P, Sun D, Yang J. Interaction between periodontitis and liver diseases. Biomed Rep. 2016;5(3):267–76. 27588170 10.3892/br.2016.718 PMC4998044

[15] Ponziani FR, Bhoori S, Castelli C, Putignani L, Rivoltini L, Del Chierico F, et al. Hepatocellular carcinoma is associated with gut microbiota profile and inflammation in non-alcoholic fatty liver disease. Hepatology. 2019;69(1):107–20. 29665135 10.1002/hep.30036

[16] Akinkugbe AA, Barritt AS, Cai J, Offenbacher S, Thyagarajan B, Khambaty T, et al. Periodontitis and non-alcoholic fatty liver disease, a population-based cohort investigation in the study of health in Pomerania. J Clin Periodontol. 2017;44(11):1077–87. 28804947 10.1111/jcpe.12800 PMC5650532

[17] Alazawi W, Bernabe E, Tai D, Janicki T, Kemos P, Samsuddin S, et al. Periodontitis is associated with significant hepatic fibrosis in patients with non-alcoholic fatty liver disease. PLoS ONE. 2017;12(12):e0185902. 29220367 10.1371/journal.pone.0185902 PMC5722374

[18] Rinčić G, Gaćina P, VirovićJukić L, Rinčić N, Božić D, Badovinac A. Association between periodontitis and liver disease. Acta Clin Croat. 2022;60(3):510–8. 35282488 10.20471/acc.2021.60.03.22 PMC8907939

[19] Yeh M, Brunt E. Pathological features of fatty liver disease. Gastroenterology. 2014;147(4):754–64. 25109884 10.1053/j.gastro.2014.07.056

[20] Diehl AM, Day C. Cause, pathogenesis, and treatment of nonalcoholicsteatohepatitis. N Engl J Med. 2017;377:2063–72. 29166236 10.1056/NEJMra1503519

[21] Alaa1 H, Mahmoud A, Dalia S, Somaia S, Sheren Y, Mohammed E. Med J Viral Hepatitis. 2019;3: 65–74.***

[22] Shiha G, Mousa N. Non-alcoholic steatohepatitis or metabolic-associated fatty liver: time to change. Hepatobiliary Surg Nutr. 2021;10(1):123–5. 33575301 10.21037/hbsn-20-438 PMC7867717

[23] Eslam M, Sanyal AJ, George J, et al. MAFLD: a consensus-driven proposed nomenclature for metabolic associated fatty liver disease. Gastroenterology. 2020;158:1999–2014. 32044314 10.1053/j.gastro.2019.11.312

[24] Hagström H, Vessby J, Ekstedt M, et al. 99% of patients with NAFLD meet MASLD criteria and natural history is therefore identical. J Hepatol. 2023;80:e76–7. 37678723 10.1016/j.jhep.2023.08.026

[25] Raj R. Metabolic associated fatty liver disease as the new nomenclature for nonalcoholic fatty liver disease. Med J Viral Hepatitis. 2022;6(2):4–5.

[26] De A, Bhagat N, Mehta M, Taneja S, Duseja A. Metabolic Dysfunction-Associated Steatotic Liver Disease (MASLD) definition is better than MAFLD criteria for lean patients with NAFLD. J Hepatol. 2024;80(2):e61–2. 37558135 10.1016/j.jhep.2023.07.031

[27] Akinkugbe AA, Avery CL, Barritt AS, et al. Do genetic markers of inflammation modify the relationship between periodontitis and non-alcoholic fatty liver disease? Findings from the SHIP study. J Dent Res. 2017;96:1392–9. 28732187 10.1177/0022034517720924 PMC5652859

[28] Kuroe K, Furuta M, Takeuchi K, et al. Association between periodontitis and fibrotic progression of non-alcoholic fatty liver among Japanese adults. J Clin Periodontol. 2021;48:368–77. 33368494 10.1111/jcpe.13415

[29] Helenius-Hietala J, Suominen AL, Ruokonen H, et al. Periodontitis is associated with incident chronic liver disease-a population- based cohort study. Liver Int. 2019;39:583–91. 30300961 10.1111/liv.13985

[30] Ahmad A, Furuta M, Shinagawa T, Takeuchi K, Takeshita T, Shimazaki, et al. Association of periodontal status with liver abnormalities and etabolicsyndrome. J Oral Sci. 2015;57:335–43. 26666857 10.2334/josnusd.57.335

[31] Kim JY, Lee GN, Song HC, Park YM, Ahn YB, Han K. Ko, SH association between fatty liver index and periodontitis: the Korea National Health and Nutrition Examination Survey. Sci Rep. 2020;10:3805. 32123238 10.1038/s41598-020-60797-7 PMC7051950

[32] Shin HS. Association between periodontal status and non-alcoholic fatty liver disease in a Korean adult population: a nationwide cross-sectional study. J Periodontol. 2020;91:524–32. 31484207 10.1002/JPER.19-0291

[33] Alazawi W, Bernabe E, Tai D, et al. Periodontitis is associated with significant hepatic fibrosis in patients with non- alcoholic fatty liver disease. PLoS ONE. 2017;12:e0185902. 29220367 10.1371/journal.pone.0185902 PMC5722374

[34] Chen Y, Yang YC, Zhu BL, Wu CC, Lin RF, Zhang X. Association between periodontal disease, tooth loss and liver diseases risk. J Clin Periodontol. 2020;47:1053–63. 32621350 10.1111/jcpe.13341

[35] Wijarnpreecha K, Panjawatanan P, Cheungpasitporn W, Lukens FJ, Harnois DM, Pungpapong S, et al. The association between periodontitis and non-alcoholic fatty liver disease: a systematic review and meta-analysis. J Gastrointest Liver Dis. 2020;29:211–7. 10.15403/jgld-841 32530988

[36] Lamont RJ, Hajishengallis G. Polymicrobial synergy and dysbiosis in inflammatory disease. Trends Mol Med. 2015;21(3):172–83. 25498392 10.1016/j.molmed.2014.11.004 PMC4352384

[37] Lynge Pedersen AM, Belstrøm D. The role of natural salivary defences in maintaining a healthy oral microbiota. J Dent. 2019;80(Suppl1):S3–12. 30696553 10.1016/j.jdent.2018.08.010

[38] Farias BC, Souza PR, Ferreira B, Melo RS, Machado FB, Gusmão ES, et al. Occurrence of periodontal pathogens among patients with chronic periodontitis. Braz J Microbiol. 2012;43(3):909–16. 24031906 10.1590/S1517-83822012000300009 PMC3768898

[39] Koren O, Spor A, Felin J, Fåk F, Stombaugh J, Tremaroli V, et al. Human oral, gut, and plaque microbiota in patients with atherosclerosis. Proc Natl Acad Sci USA. 2011;108(Suppl 1):4592–8. 20937873 10.1073/pnas.1011383107 PMC3063583

[40] Lou J, Jiang Y, Rao B, Li A, Ding S, Yan H, et al. Fecalmicrobiomes distinguish patients with autoimmune hepatitis from healthy individuals. Front Cell Infect Microbiol. 2020;10:342. 32850468 10.3389/fcimb.2020.00342 PMC7416601

[41] Mousa E, Rashed HR. Is there is a link between Oral microbiome and chronic liver diseases. Med J Viral Hepatitis. 2022;6(3):19–21.

[42] Kardesler L, Buduneli N, Cetinkalp S, Kinane DF. Adipokines and inflammatory mediators after initial periodontal treatment in patients with type 2 diabetes and chronic periodontitis. J Periodontol. 2010;81:24–33. 20059414 10.1902/jop.2009.090267

[43] Goncalves TE, Zimmermann GS, Figueiredo LC, et al. Local and serum levels of adipokines in patients with obesity after periodontal therapy: one-year follow-up. J Clin Periodontol. 2015;42:431–9. 25858047 10.1111/jcpe.12396

[44] Saito T, Shimazaki Y, Koga T, Tsuzuki M, Ohshima A. Relationship between periodontitis and hepatic condition in Japanese women. J Int Acad Periodontol. 2006;8(3):89–95. 16865998

[45] Amazaki K, Kato T, Tsuboi Y, et al. Oral pathobiont-induced changes in gut microbiota aggravate the pathology of Non-alcoholic Fatty Liver Disease in mice. Front Immunol. 2021;11(12):766170. 10.3389/fimmu.2021.766170 PMC8543001 34707622

[46] Kuraji R, Sekino S, Kapila Y, Numabe Y. Periodontal disease-related non-alcoholic fatty liver disease and nonalcoholicsteatohepatitis: an emerging concept of oral-liver axis. Periodontol 2000. 2021;87(1):204–40. 34463983 10.1111/prd.12387 PMC8456799

[47] Fiorillo L, Cervino G, Laino L, D’ Amico C, Mauceri R, Tozum TF, et al. Porphyromonas gingivalis, periodontal and systemic implications: a systematic review. Dent J. 2019;7:114. 10.3390/dj7040114 PMC6960968 31835888

[48] Sato K, Takahashi N, Kato T, Matsuda Y, Yokoji M, Yamada M, et al. Aggravation of collagen-induced arthritis by orally administered *Porphyromonas gingivalis* through modulation of the gut microbiota and gut immune system. Sci Rep. 2017;7(1):6955. 28761156 10.1038/s41598-017-07196-7 PMC5537233

[49] Furusho H, Miyauchi M, Hyogo H, Inubushi T, Ao M, Ouhara K, et al. Dental infection of *Porphyromonas gingivalis* exacerbates high fat diet-induced steatohepatitis in mice. J Gastroenterol. 2013;48:1259–70. 23307045 10.1007/s00535-012-0738-1

[50] Mulhall H, Huck O, Amar S. *Porphyromonas gingivalis*, a long-range pathogen: systemic impact and therapeutic implications. Microorganisms. 2020;8:869. 32526864 10.3390/microorganisms8060869 PMC7357039

[51] Jain P, Hassan N, Khatoon K, Mirza MA, Naseef PP, Kuruniyan MS, et al. Periodontitis and systemic disorder-an overview of relation and novel treatment modalities. Pharmaceutics. 2021;13(8):1175. 34452136 10.3390/pharmaceutics13081175 PMC8398110

[52] Paul O, Arora P, Mayer M, Chatterjee S. Inflammation in periodontal disease: possible link to vascular disease. Front Physiol. 2021;11:1818. 10.3389/fphys.2020.609614 PMC7841426 33519515

[53] Del Campo JA, Gallego P, Grande L. Role of inflammatory response in liver diseases: therapeutic strategies. World J Hepatol. 2018;10(1):1–7. 29399273 10.4254/wjh.v10.i1.1 PMC5787673

[54] Albillos A, Lario M, Álvarez-Mon M. Cirrhosis-associated immune dysfunction: distinctive features and clinical relevance. J Hepatol. 2014;61(6):1385–96. 25135860 10.1016/j.jhep.2014.08.010

[55] Hatasa M, Yoshida S, Takahashi H, Tanaka K, Kubotsu Y, Ohsugi Y, et al. Relationship between NAFLD and Periodontal Disease from the View of Clinical and Basic Research, and Immunological Response. Int J Mol Sci. 2021;22(7):3728. 33918456 10.3390/ijms22073728 PMC8038294

[56] Jepsen S, Caton JG, Albandar JM, et al. Periodontal manifestations of systemic diseases and developmental and acquired conditions: consensus report of workgroup 3 of the 2017 World Workshop on the Classification of Periodontal and Peri-Implant Diseases and Conditions. J Clin Periodontol. 2018;45(S20):S219–29. 29926500 10.1111/jcpe.12951

[57] Saito T, Shimazaki Y, Sakamoto M. Obesity and periodontitis. N Engl J Med. 1998;339(7):482–3. 9705695 10.1056/NEJM199808133390717

[58] Wood N, Johnson RB, Streckfus CF. Comparison of body composition and periodontal disease using nutritional assessment techniques: third National Health and Nutrition Examination Survey (NHANES III). J Clin Periodontol. 2003;30(4):321–7. 12694430 10.1034/j.1600-051x.2003.00353.x

[59] Benova A, Tencerova M. Obesity-induced changes in bone marrow homeostasis. Front Endocrinol. 2020;11:294. 10.3389/fendo.2020.00294 PMC7235195 32477271

[60] Eaimworawuthikul S, Thiennimitr P, Chattipakorn N, Chattipakorn SC. Diet-induced obesity, gut microbiota and bone, including alveolar bone loss. Arch Oral Biol. 2017;78:65–81. 28213172 10.1016/j.archoralbio.2017.02.009

[61] Damanaki A, Memmert S, Nokhbehsaim M, Sanyal A, Gnad T, Pfeifer A, et al. Impact of obesity and aging on crestal alveolar bone height in mice. Ann Anat. 2018;218:227–35. 29730468 10.1016/j.aanat.2018.04.005

[62] López-Gómez JJ, Pérez Castrillón JL, de Luis-Román DA. Impact of obesity on bone metabolism. Endocrinol Nutr. 2016;63:551–9. 27744014 10.1016/j.endonu.2016.08.005

[63] Yu T, Zhao L, Huang X, Xie B, Zhang J, Xuan D. Aberrant periodontal and systemic immune response of overweight rodents to periodontal infection. Biomed Res Int. 2019;3:2019. 10.1155/2019/9042542 PMC6335672 30719451

[64] Levine RS. Obesity, diabetes and periodontitis–a triangular relationship? Br Dent J. 2013;215(1):35–9. 23846063 10.1038/sj.bdj.2013.627

[65] Preshaw PM, Alba AL, Herrera D, et al. Periodontitis and diabetes: a two-way relationship. Diabetologia. 2012;55:21–31. 22057194 10.1007/s00125-011-2342-y PMC3228943

[66] Chapple IL, Genco R, Working group 2 of the Joint EFP/AAP Workshop. Diabetes and periodontal diseases: consensus report of the joint EFP/AAP workshop on periodontitis and systemic diseases. J Periodontol. 2013;84:S106–12. 23631572 10.1902/jop.2013.1340011

[67] Preshaw P, Bissett S. Periodontitis and diabetes. Br Dent J. 2019;227:577–84. 31605062 10.1038/s41415-019-0794-5

[68] Tsai C, Hayes C, Taylor GW. Glycaemic control of type 2 diabetes and severe periodontal disease in the US adult population. Community Dent Oral Epidemiol. 2002;30:182–92. 12000341 10.1034/j.1600-0528.2002.300304.x

[69] Oberti L, Gabrione F, Nardone M, Di Girolamo M. Two-way relationship between diabetes and periodontal disease: a reality or a paradigm? J Biol Regul Homeost Agents. 2019;33(Suppl. 1):153–9. 31538462

[70] Chopra A, Jayasinghe TN, Eberhard J. Are inflamed periodontal tissues endogenous source of Advanced Glycation End-Products (AGEs) in individuals with and without diabetes mellitus? A systematic review. Biomolecules. 2022;12(5):642. 35625570 10.3390/biom12050642 PMC9138899

[71] Allen EM, Matthews JB, Griffiths HR, Chapple IL. Oxidative and inflammatory status in Type 2 diabetes patients with periodontitis. J Clin Periodontol. 2011;38:894–901. 21883360 10.1111/j.1600-051X.2011.01764.x

[72] Bastos AS, Graves DT, Loureiro AP, Rossa Junior C, Abdalla DS, FaulinTdo E, et al. Lipid peroxidation is associated with the severity of periodontal disease and local inflammatory markers in patients with type 2 diabetes. J Clin Endocrinol Metab. 2012;97:E1353-1362. 22564665 10.1210/jc.2011-3397 PMC3410275

[73] Llambés F, Arias-Herrera S, Caffesse R. Relationship between diabetes and periodontal infection. World J Diabetes. 2015;6(7):927–35. 26185600 10.4239/wjd.v6.i7.927 PMC4499526

[74] Santana RB, Xu L, Chase HB, Amar S, Graves DT, Trackman PC. A role for advanced glycation end products in diminished bone healing in type 1 diabetes. Diabetes. 2003;52:1502–10. 12765963 10.2337/diabetes.52.6.1502

[75] Cortizo AM, Lettieri MG, Barrio DA, Mercer N, Etcheverry SB, McCarthy AD. Advanced glycation end-products (AGEs) induce concerted changes in the osteoblastic expression of their receptor RAGE and in the activation of extracellular signal-regulated kinases (ERK). Mol Cell Biochem. 2003;250:1–10. 12962137 10.1023/a:1024934008982

[76] Lalla E, Lamster IB, Drury S, Fu C, Schmidt AM. Hyperglycemia, glycoxidation and receptor for advanced glycation endproducts: potential mechanisms underlying diabetic complications, including diabetes-associated periodontitis. Periodontol 2000. 2000;23:50–62. 11276765 10.1034/j.1600-0757.2000.2230104.x

[77] Polak D, Shapira L. An update on the evidence for pathogenic mechanisms that may link periodontitis and diabetes. J Clin Periodontol. 2018;45(2):150–66. 29280184 10.1111/jcpe.12803

[78] Aimetti M, Perotto S, Castiglione A, Mariani GM, Ferrarotti F, Romano F. Prevalence of periodontitis in an adult population from an urban area in North Italy: findings from a cross-sectional population-based epidemiological survey. J Clin Periodontol. 2015;42:622–31. 25970460 10.1111/jcpe.12420

[79] Landi L, Grassi G, Sforza MN, Ferri C. Hypertension and periodontitis: an upcoming joint report by the Italian Society of Hypertension (SIIA) and the Italian Society of Periodontology and Implantology (SIdP). High Blood Press Cardiovasc Prev. 2021;28:1–3. 33400213 10.1007/s40292-020-00430-w

[80] Sanz M, Marco Del Castillo A, Jepsen S, Gonzalez-Juanatey JR, D’Aiuto F, Bouchard P, et al. Periodontitis and cardiovascular diseases: consensus report. J ClinPeriodontol. 2020;47:268–88. 10.1111/jcpe.13189 PMC7027895 32011025

[81] Del Pinto R, Pietropaoli D, Munoz-Aguilera E, D’Aiuto F, Czesnikiewicz-Guzik M, Monaco A, et al. Periodontitis and hypertension: is the association causal? High Blood Press Cardiovasc Prev. 2020;27:281–9. 32500479 10.1007/s40292-020-00392-z

[82] Aguilera E, Suvan J, Buti J, et al. Periodontitis is associated with hypertension: a systematic review and meta-analysis. Cardiovasc Res. 2020;116:28–39. 31549149 10.1093/cvr/cvz201

[83] Surma S, Romańczyk M, Witalińska-Łabuzek J, et al. Periodontitis, blood pressure, and the risk and control of arterial hypertension: epidemiological, clinical, and pathophysiological aspects—review of the literature and clinical trials. Curr Hypertens Rep. 2021;23:27. 33961166 10.1007/s11906-021-01140-x PMC8105217

[84] Rosa RAC, Rodrigues JVS, Cláudio MM, Franciscon JPS, Mulinari-Santos G, Cirelli T, et al. The relationship between hypertension and periodontitis: a cross-sectional study. J Clin Med. 2023;12(15):5140. 37568542 10.3390/jcm12155140 PMC10419474

[85] Al-Ghurabei BH. Evaluation of serum anti-cardiolipin antibody, hs-CRP and IL-6 levels in chronic periodontitis as possible risk factors for cardiovascular diseases. J Baghdad Coll Dent. 2012;24(2):161–5.

[86] Pignatelli P, Fabietti B, Ricci A, Piattelli A, Curia M. How periodontal disease and presence of nitric oxide reducing oral bacteria can affect blood pressure. Int J Mol Sci. 2020;21:7538. 33066082 10.3390/ijms21207538 PMC7589924

[87] Law MR, Morris JK, Wald NJ. Use of blood pressure lowering drugs in the prevention of cardiovascular disease: meta-analysis of 147 randomised trials in the context of expectations from prospective epidemiological studies. BMJ. 2009;19(338):b1665. 10.1136/bmj.b1665 PMC2684577 19454737

[88] Guzik TJ, Skiba DS, Touyz RM, Harrison DG. The role of infiltrating immune cells in dysfunctional adipose tissue. Cardiovasc Res. 2017;113:1009–23. 28838042 10.1093/cvr/cvx108 PMC5852626

[89] Mikolajczyk TP, Nosalski R, Szczepaniak P, Budzyn K, Osmenda G, Skiba D, et al. Role of chemokine RANTES in the regulation of perivascular inflammation, T-cell accumulation, and vascular dysfunction in hypertension. FASEB J. 2016;30:1987–99. 26873938 10.1096/fj.201500088R PMC4836375

[90] Cutler CW, Shinedling EA, Nunn M, Jotwani R, Kim BO, Nares S, et al. Association between periodontitis and hyperlipidemia: cause or effect? J Periodontol. 1999;70(12):1429–34. 10632517 10.1902/jop.1999.70.12.1429

[91] Taleghani F, Shamaei M, Shamaei M. Association between chronic periodontitis and serum lipid levels. Acta Med Iran. 2010;48(1):47–50. 21137669

[92] Kinane DF. Periodontal diseases’ contributions to cardiovascular disease: an overview of potential mechanisms. Ann Periodontol. 1998;3:142–50. 9722698 10.1902/annals.1998.3.1.142

[93] Naock B, Jachmann I, Roscher S, Sieber L, Kopprasch S, Lück C, et al. Metabolic diseases and their possible link to risk indicators of periodontitis. J Periodontol. 2000;71:898–903. 10914792 10.1902/jop.2000.71.6.898

[94] Katz J, Chaushu G, Sharabi V. On the association between hypercholesterolemia, cardiovascular disease and severe periodontal disease. J Clin Periodontol. 2001;28:865–8. 11493357 10.1034/j.1600-051x.2001.028009865.x

[95] Iacopino AM, Cutler CW. Path physiologic relationships between periodontitis and systemic diseases: recent concept involving serum lipids. J Periodontal. 2000;71(8):1375–84. 10.1902/jop.2000.71.8.1375 10972656

[96] Khovidhunkit W, Kim MS, Memon RA, Shigenaga JK, Moser AH, Feingold KR, et al. Effects of infection and inflammation on lipid and lipoprotein metabolism: mechanisms and consequences to the host. J Lipid Res. 2004;45(7):1169–96. 15102878 10.1194/jlr.R300019-JLR200

[97] Hudgins LC, Parker TS, Levine DM, Gordon BR, Saal SD, Jiang X-C, et al. A single intravenous dose of endotoxin rapidly alters serum lipoproteins and lipid transfer proteins in normal volunteers. J Lipid Res. 2003;44(8):1489–98. 12754273 10.1194/jlr.M200440-JLR200

[98] Iacopino AM, Cutler CW. Pathophysiological relationships between periodontitis and systemic disease: recent concepts involving serum lipids. J Periodontol. 2000;71:1375–84. 10972656 10.1902/jop.2000.71.8.1375

[99] Song TJ, Kim JW, Kim J. Oral health and changes in lipid profile: a nationwide cohort study. J Clin Periodontol. 2020;47(12):1437–45. 32996160 10.1111/jcpe.13373

[100] Harrison SA, Bedossa P, Guy CD, Schattenberg JM, Loomba R, Taub R, et al. A phase 3, randomized, controlled trial of resmetirom in nash with liver fibrosis. N Engl J Med. 2024;390(6):497–509. 38324483 10.1056/NEJMoa2309000

[101] Abou Assi R, Abdulbaqi IM, Siok YC. The evaluation of drug delivery nanocarrier development and pharmacological briefing for metabolic-associated fatty liver disease (MAFLD): an update. Pharmaceuticals. 2021;14:215. 33806527 10.3390/ph14030215 PMC8001129

[102] Gameil M, Rozaik S, Ahmed Elsebaie A, Marzouk R. Influence of liraglutide, dulaglutide versus conventional treatment on fatty liver index and fibrosis-4 score in Egyptian patients with type 2 diabetes mellitus and non-alcoholic fatty liver disease. Med J Viral Hepat. 2018;5(1):25–32.

[103] Nayak IN, Narendar K, Jamadar M, Kumar VH. Comparison of pioglitazone and metformin efficacy against glucocorticoid induced atherosclerosis and hepatic steatosis in insulin resistant rats. J Clin Diagn Res. 2017;11:FC06. 28892924 10.7860/JCDR/2017/28418.10193 PMC5583870

